# Assessment of the Antimicrobial Activity of *Cistus salviifolius* L. and *Helichrysum stoechas* (L.) DC Extracts and Their Synergistic Potential with Conventional Antibiotics Against *Staphylococcus aureus*

**DOI:** 10.3390/ijms262311331

**Published:** 2025-11-24

**Authors:** Alexandra Coimbra, Ângelo Luís, Pedro Dinis Gaspar, Susana Ferreira, Ana Paula Duarte

**Affiliations:** 1 RISE-Health, Department of Medical Sciences, Faculty of Health Sciences, University of Beira Interior, Av. Infante D. Henrique, 6200-506 Covilhã, Portugal; alexandra.coimbra@ubi.pt (A.C.); angelo.luis@ubi.pt (Â.L.); susana.ferreira@fcsaude.ubi.pt (S.F.); 2 C-MAST—Center for Mechanical and Aerospace Science and Technologies, University of Beira Interior, 6201-001 Covilhã, Portugal; dinis@ubi.pt

**Keywords:** *Cistus salviifolius*, *Helichrysum stoechas*, antimicrobial agent, *Staphylococcus aureus*, virulence, adjuvant, quorum sensing, biofilm

## Abstract

This study aimed to evaluate the antibacterial activity of *Cistus salviifolius* L. and *Helichrysum stoechas* (L.) DC extracts against *S. aureus*, including methicillin-resistant *S. aureus* (MRSA) strains. To this end, assays were conducted to assess killing kinetics, antibiotic combination effects, modulatory effects on ethidium bromide, inhibition of quorum sensing, and biofilm formation. *H. stoechas* extract demonstrated the strongest activity, with MIC values ranging from 7.8 to 62.5 µg/mL. When combined with antibiotics such as ampicillin, ciprofloxacin, or vancomycin, the extracts of *C. salviifolius* and *H. stoechas* predominantly exhibited synergistic (FICI value ≤ 0.5) or additive effects (0.5 < FICI ≤ 1), with some combinations resensitizing resistant strains. The aerial parts of *C. salviifolius* displayed modulatory effects on ethidium bromide MIC, reducing the concentration from 32 to 8 µg/mL, suggesting efflux pump inhibitory activity. In addition, this extract displayed slight quorum-sensing inhibition at a concentration of 125 µg/mL. Moreover, *C. salviifolius* and *H. stoechas* extracts inhibit the formation of biofilm by *S. aureus* strains, even at subinhibitory concentrations (0.5× and 0.25× MIC). The presence of compounds such as myricetin 3 *O*-galactoside, catechin derivatives, gallic acid, kaempferol, and chlorogenic acid in the extracts may contribute to their anti-*Staphylococcus* activity. These results demonstrated the dual antimicrobial and antivirulence potential of *C. salviifolius* and *H. stoechas* extracts, highlighting their promise as therapeutic agents or adjuvants against *S. aureus.* These extracts can be promising candidates for further studies on the development of novel strategies targeting multiple pathogenic pathways.

## 1. Introduction

*Staphylococcus aureus* is a Gram-positive bacterium with a spherical shape that typically forms clusters [1]. Typically a commensal organism, *S. aureus* can become an opportunistic pathogen capable of causing diverse and severe infections. It commonly colonizes humans, most frequently affecting the skin and soft tissues; however, it is also one of the leading opportunistic bacterial pathogens, responsible for significant morbidity and mortality worldwide [1,2,3,4,5].

The development and use of antibiotic therapies have markedly reduced mortality associated with *S. aureus* infections; however, this has been accompanied by the emergence and proliferation of multidrug-resistant strains [6]. The acquisition of resistance has driven the worldwide spread of diverse methicillin-resistant *S. aureus* (MRSA) lineages [2]. The rapid adaptive capacity of MRSA, coupled with the accumulation of additional acquired resistance mechanisms, further exacerbates the challenges in the effective management and therapeutic control of *S. aureus* infections [7]. In such a manner, MRSA antibiotic resistance poses a serious clinical challenge, and it is among the most pressing and urgent threats to global public health nowadays [6,8,9,10].

The emergence of antibiotic resistance currently exceeds the rate at which new drugs are developed for treating infections [10,11]. The development of new antibiotics is often not cost-effective, leading to a shortage of novel drugs and further aggravating the problem of resistance management [12,13,14]. Among different strategies, antibiotic combination has been shown to substantially enhance antibacterial efficacy in the treatment of multi-drug-resistant *S. aureus* infection [6,12,13,14]. In the context of increasing antimicrobial resistance, novel strategies are being explored, including the use of plant-derived compounds as antimicrobial agents, anti-virulence modulators, or adjuvants that enhance the efficacy of conventional antibiotics. Among these approaches, antibiotic adjuvants represent a particularly promising and complementary strategy to combat antibiotic resistance by either directly targeting resistance mechanisms or enhancing the activity of existing antibiotics, thereby restoring or improving their antimicrobial effectiveness [10,12]. Commonly used antibiotic adjuvants include β-lactamase inhibitors, efflux pump inhibitors, and outer membrane permeabilizers, each targeting specific bacterial resistance mechanisms [12]. Another promising approach is the use of anti-virulence strategies as adjuvants to antibiotic therapy [10]. Anti-virulence compounds can be particularly advantageous, as they may attenuate pathogenicity without compromising bacterial viability, thereby reducing selective pressure and minimizing the risk of resistance development [10].

Herbal medicines have been used for centuries to treat infectious diseases, and growing evidence indicates that plant extracts exhibit notable activity against microorganisms, including drug-resistant strains such as MRSA strains [15]. Consequently, plant-derived compounds are being recognized as promising candidates for the development of novel antimicrobial strategies [14,16,17]. Plants have evolved sophisticated defense mechanisms against microbial pathogens, including production of enzymes and secondary metabolites, reinforcement of cellular structures, and other protective strategies against environmental stressors and predators [14,18]. These natural defenses have provided considerable therapeutic potential. While traditional remedies relied on crude extracts or powdered preparations, modern approaches have refined plant-based drugs into purified phytochemicals with significant pharmacological properties [19]. Among these, polyphenol-rich plant extracts have demonstrated inhibitory effects against pathogenic bacteria and fungi, with both whole extracts and isolated compounds, showing activity against *S. aureus* [1,14,19,20,21,22].

Plant species of the genus *Cistus* L. (family Cistaceae) are evergreen flowering shrubs, commonly known as rock-roses, primarily distributed across the Mediterranean region. They thrive in harsh environments, including rocky, dry, semi-arid, or nutrient-poor soils, and have a long history of use in traditional folk medicine for various purposes [23,24,25,26]. Most species of this family, characterized by their fragrance and sweet scent, are also highly valued in the perfume industry [26]. *Cistus salviifolius* L. is a perennial shrub reaching up to 60 cm in height, with ovate-elliptic, small, simple, and rough-textured leaves, and actinomorphic white flowers. Its flowering period typically spans from March to May [24,27,28]. Extracts of *C. salviifolius* have been shown to exhibit various bioactive properties, including antioxidant, anti-inflammatory, anti-acetylcholinesterase, anti-xanthine oxidase, antidiabetic, antihypertensive, anticancer, antiproliferative, and antimicrobial activity [24,25,26,29,30,31,32,33,34,35,36,37,38,39,40,41].

The genus *Helichrysum* Mill., belonging to the Asteraceae family, includes up to 600 species of flowering plants widely distributed across the southern regions of the world. Species within this genus may be annuals, herbaceous perennials, or shrubs, with some reaching heights of up to 90 cm [38,42,43,44]. *Helichrysum stoechas* (L.) DC, commonly known as Mediterranean strawflower, curry plant, or yellow amaranth, is a fragrant, thermophilic halophyte native to southern Europe. It is an evergreen aromatic subshrub, either perennial or annual, that thrives in dry, rocky, and sandy soils. The plant is hermaphroditic, with grayish-green foliage and small, spherical yellow inflorescences [38,45]. Extracts of *H. stoechas* also display bioactive properties, particularly antioxidant, antiviral, antiproliferative, antidiabetic, neuroprotective, anti-inflammatory, antihypertensive, analgesic, anticancer, and antimicrobial activity [41,42,43,46,47,48,49,50,51,52].

Despite previous studies reporting the biological activities of *C. salviifolius* and *H. stoechas*, limited information is available regarding their antibacterial and antivirulence properties against *S. aureus*, including MRSA. Moreover, the potential synergistic interactions of these extracts with conventional antibiotics and their effects on key virulence mechanisms, such as quorum sensing and biofilm formation, remain poorly understood. Therefore, this study aimed to address these knowledge gaps by evaluating the antimicrobial of *C. salviifolius* and *H. stoechas* and assessing their impact on the bacterium’s virulence and investigate their combined effect with conventional antibiotics.

## 2. Results and Discussion

In our previous study [41], we conducted a broad screening of extracts from seven different plant species to evaluate their bioactive potential, including antioxidant capacity, in vitro anti-inflammatory effects, antimicrobial activity, biocompatibility, and chemical composition. From this initial assessment, *C. salviifolius* and *H. stoechas* emerged as the most promising candidates, particularly due to their notable antimicrobial effects. Since the strongest activity was observed against Gram-positive bacteria—most prominently *S. aureus*—we selected *S. aureus* for a more detailed investigation.

### 2.1. Phytochemical Characterization

The preparation of the hydroethanolic extracts of *C. salviifolius* aerial parts and stems, and of *H. stoechas,* as well as their phytochemical analysis, has been described in previous work [41]. Briefly, *C. salviifolius* extracts were rich in flavonoids and phenolic acids, particularly neochlorogenic acid, gallic acid, gallocatechin 3-*O*-gallate, and rutin and arabinoside derivatives as quercetin glycosides. They are also characterized by the presence of kaempferol 3-*O*-glucosyl-rhamnosyl-galactoside and additional coumarins, including scopoletin and coumarin. *H. stoechas* extract showed the presence of hydroxycinnamic acid esters, particularly 3-feruloylquinic acid and 3,4-dicaffeoylquinic acid, as well as chlorogenic and neochlorogenic acids. In the case of 3,4-dicaffeoylquinic acid, this compound was identified exclusively in the extract of *H. stoechas*. A range of flavonoids, including myricetin, quercetin, and luteolin glycosides, were also detected, along with scopoletin and usnic acid [41].

### 2.2. Activity of Cistus salviifolius and Helichrysum stoechas Extracts Against S. aureus Strains

Considering the documented antimicrobial potential of the *C. salviifolius* and *H. stoechas* extracts and their profile as polyphenol-rich extracts, their activity against *S. aureus* was further explored. The evaluation was performed using a reference strain (*S. aureus* ATCC 25923) and two methicillin-resistant clinical isolates (MRSA 12/08 and MRSA 05/15), by first determining the minimum inhibitory concentrations (MICs) of the extracts (Table 1).

The extract with the most promising anti-staphylococcal activity was from *H. stoechas*, exhibiting similar antimicrobial activity to the antibiotic vancomycin. Regarding the MRSA 12/08, the MIC values obtained for the extracts increased compared to the other strains, with the extract obtained from the stems of *C. salviifolius* showing no activity in the tested range of concentrations and thus showing the influence of plant parts on the antibacterial activity.

The antibacterial activity of *C. salviifolius* extracts against *S. aureus* strains aligns with previous studies [24,25,29,30,31,32,33,34,35,36,37], though reported efficacy varies due to plant growth conditions, extraction methods, and experimental conditions [53,54]. *C. salviifolius* aqueous extracts activity has been linked to antibiotic resistance profiles of *S. aureus* clinical isolates, with β-lactams and quinolones resistance strains showing higher susceptibility. Mechanistically, disruption of the bacterial plasma membrane or cell wall has been proposed as a possible mode of action [30]. Polyphenols appear to play a central role in the observed effects, as in MRSA, molecules bearing COOH and OH groups in *ortho* and *para* positions or an O–CH_3_ group in the *meta* position have been associated with enhanced anti-MRSA activity [55], a feature consistent with the flavonoids identified in our extracts. Synergistic interactions among major polyphenols of *C. salviifolius* can enhance activity (up to 20-fold) compared to individual compounds, in some cases approaching that of standard antibiotics. Myricetin was identified as the main contributor to these effects [56], since our extracts contain myricetin-3-*O*-galactoside, a glycosylated form of myricetin, this compound may significantly contribute to the observed anti-staphylococcal activity.

For *H. stoechas* extracts, evidence suggests strong activity against Gram-positive bacteria, particularly *S. aureus*, with its efficacy being influenced by extraction solvents, with MICs ranging from 8 to 250 µg/mL in Gram-positive bacteria [42,43,46,47,57,58]. The chemical basis for this activity appears to be related to phenolic compounds, with dimethyl phthalate identified as an active constituent that disrupts *S. aureus* cellular structures, membrane integrity, and energy metabolism, ultimately leading to cell death [59]. Collectively, these findings indicate that *H. stoechas* possesses promising anti-staphylococcal potential.

**Figure 1:**
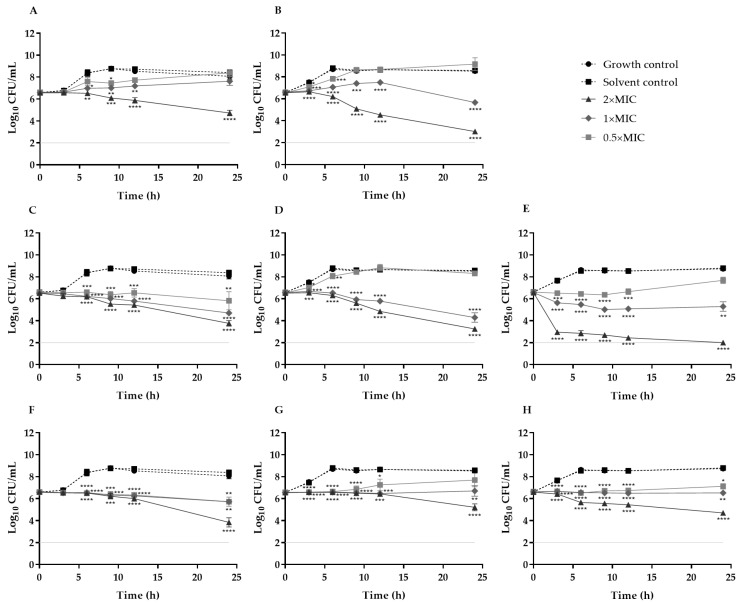
Time–kill curves for *S. aureus* ATCC 25923 (**A**,**C**,**F**), MRSA 05/15 (**B**,**D**,**G**) and MRSA 12/08 (**E**,**H**) strains incubated with *C. salviifolius* stems (**A**,**B**), *C. salviifolius* aerial parts (**C**,**D**,**E**) and *H. stoechas* (**F**,**G**,**H**) extracts from 0.5× MIC to 2× MIC at 37 °C. The light gray horizontal line at 2 Log_10_ CFU/mL represents the detection limit of the method. * (*p* < 0.05) ** (*p* < 0.01); *** (*p* < 0.001); **** (*p* < 0.0001).

To further characterize the antimicrobial properties of the plant extracts and capture their dynamics of bacterial killing, time-kill assays were performed (Figure 1). For the MRSA 12/08 strain, the stem extract of *C. salviifolius* was not included in subsequent assays, as its MIC could not be determined, thereby precluding the selection of appropriate concentrations for further methodological evaluations.

According to the classification of Ishak et al. [60], these extracts exhibited predominantly bactericidal activity (MBC/MIC ratio ≤ 4), supported by time-kill assays that revealed significant reductions in cell viability, particularly at 2× MIC, with the effect being more pronounced for *C. salviifolius*.

To our knowledge, no prior studies have evaluated the time-dependent effects of these extracts. However, in a related work with another *Helichrysum* species, exposure of *S. aureus* ATCC 6538P to *H. italicum* diethyl ether extract decreased viable counts by 3 log_10_ within 30 min at four- and ten-fold the MIC (1000 and 2500 µg/mL, respectively), with a slower killing rate after, reaching approximately ≤2 log CFU/mL after 24–48 h [61], thus underscoring the antimicrobial potential of the genus *Helichrysum* [61]. Regarding isolated compounds, gallic acid, which is present in the *C. salviifolius* and *H. stoechas* extracts, has shown notable anti-MRSA activity with a MIC value of 32 μg/mL. Growth curve analysis further revealed that at 32 and 64 μg/mL, bacterial populations remained unchanged throughout incubation, while at lower concentrations (8 and 16 μg/mL), gallic acid effectively suppressed MRSA proliferation and prolonged its growth cycle [62].

### 2.3. Combined Effects of C. salviifolius and H. stoechas Extracts and Antibiotics, and Evaluation of Modulatory Activity of These Extracts

The emergence of multidrug-resistant (MDR) pathogens has complicated the effective treatment of numerous infectious diseases [63,64]. In this context, the combination of plant-derived extracts and antibiotics has gained attention, as it can modulate efflux pump expression, disrupt cellular structures, and interfere with other resistance mechanisms in MRSA, thereby enhancing antibiotic efficacy [7,63,64]. This synergistic approach may restore antibiotic susceptibility, enable dose reduction, minimize associated toxicity, and potentially help to counteract antimicrobial resistance [13,15,63,65,66].

Accordingly, this work explored the potential synergistic interactions between *C. salviifolius* and *H. stoechas* extracts and the antibiotics ampicillin, ciprofloxacin, and vancomycin (Table 2).

The *C. salviifolius* and *H. stoechas* extracts enhanced the activity of ampicillin, ciprofloxacin, and vancomycin against the tested *S. aureus* strains, demonstrating predominantly synergistic (FICI ≤ 0.5) or additive (0.5 < FICI ≤ 1) effects. Notably, these interactions resulted in the resensitization of the resistant MRSA 12/08 strain to ampicillin or ciprofloxacin, reducing their MICs from 32 µg/mL to ≤2 µg/mL, a decrease of more than 26-fold when combined with the extracts.

The observed effects are consistent with previous reports describing the ability of catechin derivatives to act synergistically with β-lactam antibiotics [63,68,69]. Mechanistically, these compounds may enhance the activity of β-lactams by disrupting cell wall integrity, through direct binding to peptidoglycan, and by penicillinase inhibition [70]. They may also interfere with the synthesis of the penicillin-binding protein PBP2a, suppress β-lactamase secretion [71,72] and increase intracellular antibiotic accumulation potentially through the downregulation of key efflux pump genes [73]. Phytochemical analysis of the extracts further revealed that the stem-derived extract contained both (+)-gallocatechin and (+)-gallocatechin 3-*O*-gallate, whereas the *C. salviifolius* extract obtained from the aerial parts contained only (+)-gallocatechin 3-*O*-gallate. Overall, these findings highlight the potential of *C. salviifolius* extracts, particularly their (+)-gallocatechin derivatives, as adjuvants capable of restoring antibiotic efficacy and counteracting resistance mechanisms in MRSA. Concerning the compounds only present in the *H. stoechas* extract, 4′,5′-*O*-dicaffeoylquinic acid (4′,5′-*O*DCQA, an isomer of dicaffeoliquinic acid) was shown to act synergistically with fluoroquinolones against *S. aureus* [74]. Beyond these specific compounds, several compounds identified across the three extracts and their derivates also displayed synergistic interactions with β-lactam and fluroquinolones, such as quercetin, gallic acid, caffeic acid, *p*-coumaric acid, and other coumarinic compounds, and usnic acid [64,75,76,77,78,79]. Notably, ciprofloxacin–usnic acid combination exhibited synergistic or additive effects against MRSA clinical isolates, whereas the vancomycin–usnic acid combination consistently demonstrated synergy against all MRSA isolates tested [77]. Taken together, the synergistic effects observed for multiple compounds strongly suggest that the bioactive constituents of *C. salviifolius* and *H. stoechas* extracts contribute to the restoration of antibiotic activity against resistant *S. aureus* strains. These interactions are particularly relevant given the diverse resistance mechanisms of *S. aureus* [79,80,81,82,83,84].

Thus, efflux pump inhibitors (EPIs) have emerged as important modulators of antimicrobial resistance, targeting these transporters to prevent extrusion of toxic compounds, including antibiotics [80,81,82]. Medicinal plants may provide a rich source of promising EPIs, owing to their chemically and structurally diverse secondary metabolites that exhibit multiple pharmacological properties [83,84,85]. Here, the experimental approach to detect EPI activity of the extracts was to test the combined action of ethidium bromide with *C. salviifolius* and *H. stoechas* extracts added at a sub inhibitory concentration (Table 3).

A positive modulating effect was demonstrated when in the presence of aerial parts of *C. salviifolius* extract, decreasing the ethidium bromide MIC to similar levels of those observed for the positive control, reserpine, a well-known efflux pump inhibitor [83,86]. This reduction in MIC of ethidium bromide can indicate a potential inhibition of efflux pumps by the aerial parts of *C. salviifolius*. This activity may be consistent with the presence of compounds such as kaempferol glycosides, which have demonstrated potent NorA inhibition in *S. aureus*, reducing the MIC of ethidium bromide and norfloxacin by up to 64-fold and 32-fold, respectively [87,88,89]. Apigenin derivatives present in *C. salviifolius* extracts may also support these modulatory effects, as reported apigenin as an EPI [90]. Other compounds that also support efflux inhibition, are quercetin and coumarinic compounds, which by in silico analysis demonstrated to form a hydrogen bond with NorA and hydrophobic interactions, suggesting inhibition of NorA and MepA in *S. aureus,* potentially interfering with the efflux of toxic compounds like ethidium bromide [78,79,91].

Taken together, these findings indicate that the diverse mechanisms of action of compounds present in *C. salviifolius* and *H. stoechas* likely contribute, at least partially, to the enhanced anti-Staphylococcal activity of the antibiotics observed in this study beside efflux pump inhibition [92,93,94,95,96,97].

### 2.4. Inhibitory Effect of C. salviifolius and H. stoechas Extracts on Quorum Sensing

**Figure 2:**
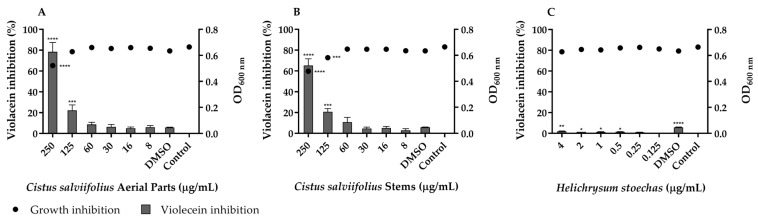
Quorum-sensing inhibition by *C. salviifolius* aerial parts (**A**), *C. salviifolius* stems (**B**) and *H. stoechas* (**C**) extracts against *Chromobacterium violaceum* ATCC 12472. The solvent control dimethyl sulfoxide (DMSO) at a final concentration of 0.5% was also tested. Percentage of violacein inhibition (%) by different concentrations of extracts and evaluation of microbial viability (OD_600 nm_) after 48 h of incubation. * (*p* < 0.05); ** (*p* < 0.01); *** (*p* < 0.001); **** (*p* < 0.0001).

Quorum sensing (QS) is a density-dependent communication mechanism that regulates bacterial gene expression, including virulence, biofilm formation, and bacterial adaptation [11,66,84,98,99,100]. Targeting QS offers a promising anti-virulence strategy, thus *Chromobacterium violaceum,* whose violacein pigment is regulated by QS [91], has been used as a biosensor to evaluate the potential of the plant extracts as QS inhibitors (Figure 2).

The extract from the aerial parts of *C. salviifolius* exhibited QS inhibitory activity at a concentration of 125 µg/mL, leading to a significant reduction in violacein production (*p* < 0.001) without affecting the growth of *C. violaceum*. In turn, the tested concentrations of *H. stoechas* did not inhibit violacein production, suggesting that this extract may not interfere with QS.

Several molecules have been identified that interfere with *S. aureus* QS. Among them, luteolin acts as a concentration-dependent antibiofilm agent against MDR *S. aureus* by inhibiting the agr QS system [101].

Simultaneously, QS signal degradation may limit their distribution within a biofilm, emphasizing the key role of efflux pumps in modulating bacterial QS responses [84]. In fact, efflux pumps can mediate the export of polymeric substances involved in biofilm production, as well as QS molecules that regulate biofilm formation, thereby influencing bacterial adhesion and aggregation on solid surfaces [102,103].

### 2.5. Inhibition of S. aureus Biofilm Formation by C. salviifolius and H. stoechas Extracts

Bacteria predominantly exist as surface-attached communities, forming biofilms on diverse biotic or abiotic surfaces [8,11,104,105]. Within biofilms, cells are embedded in a protective extracellular polymeric substance (EPS) that enhances survival, stress tolerance, and antimicrobial resistance [4,8,104,105,106,107]. Compared with planktonic cells, biofilm-associated bacteria exhibit 10–1000-fold higher tolerance to antimicrobials due to multiple factors, including restricted drug penetration, metabolic alterations, efflux pump activation, and the presence of persistent or dormant cells [11,84,104,106,107,108].

**Figure 3:**
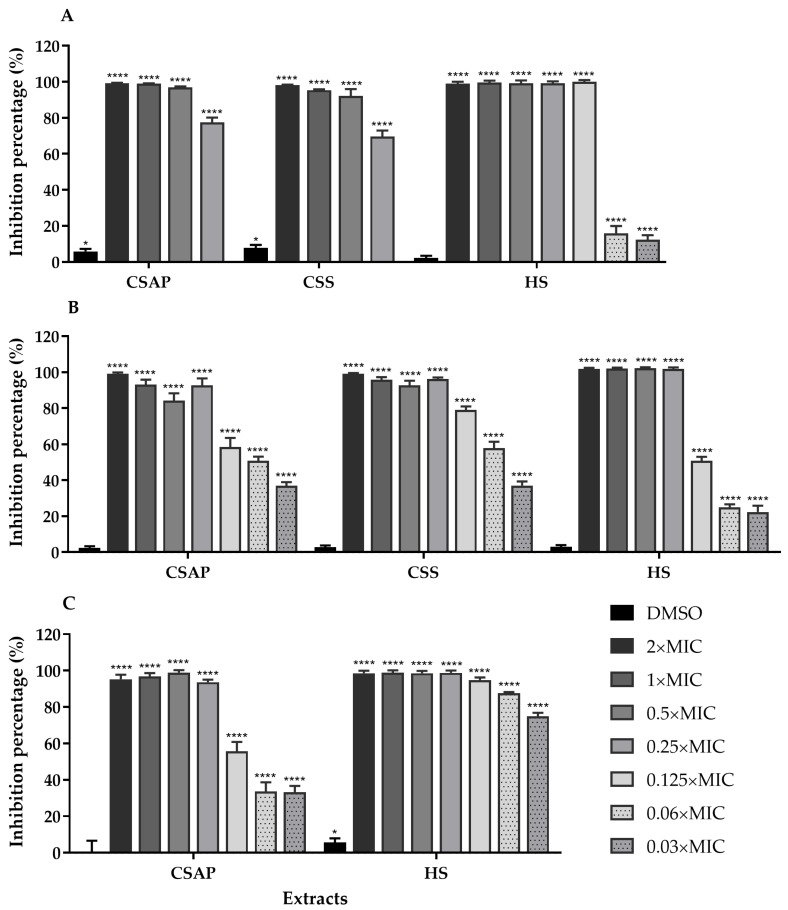
Effects of different concentrations of *C. salviifolius* aerial parts (CSAP), *C. salviifolius* stems (CSS), and *H. stoechas* (HS) extracts on the formation of biofilms of *S. aureus* ATCC 25923 (**A**), MRSA 05/15 (**B**), and MRSA 12/08 (**C**) strains. Biofilm formation was estimated by the crystal-violet assay, and results are expressed as % of biofilm biomass inhibition. Dimethyl sulfoxide (DMSO) was used as a solvent control. * (*p* < 0.05); **** (*p* < 0.0001).

Conventional physical, chemical, and antibiotic-based treatments are often ineffective or costly, underscoring the need for targeted anti-biofilm strategies, considering not only their eradication but also their formation [98,109,110]. Natural products, particularly plant-derived compounds, have emerged as promising candidates with anti-virulence and antibiofilm activity [98,99,105,108,111,112]. Accordingly, the ability of *C. salviifolius* and *H. stoechas* extracts to inhibit biofilm formation was evaluated against *S. aureus* strains (Figure 3).

The extracts of *C. salviifolius* and *H. stoechas* exhibited potent anti-virulence properties at concentrations corresponding to the MIC and 2× MIC, completely inhibiting biofilm formation in *S. aureus* strains. Remarkably, even at subinhibitory concentrations, these extracts demonstrated strong anti-biofilm activity.

The ability of *C. salviifolius* extracts to inhibit biofilm formation by *S. aureus* strains has also been demonstrated in other studies. For instance, Onal et al. [37] reported that the hydroethanolic extract of *C. salviifolius* inhibited the biofilm production of several *S. aureus* strains. A more detailed focus on compounds of the *C. salviifolius* extracts shows that (+)-gallocatechin and its derivates not only exert bactericidal effects, but also inhibit key enzymes required for *S. aureus* survival and virulence [113,114]. Similarly, quercetin and myricetin (derivatives of these compounds were identified in the three extracts) reduced the biofilm formed by different staphylococcal species and affected the formation of aggregates [115]. Other compounds also present in *H. stoechas* extracts, such as gallic acid, luteolin, myricetin, kaempferol, neochlorogenic acid, chlorogenic acid, and rutin, exhibit strong anti-biofilm activity against *S. aureus*, including MRSA, reducing the biofilm biomass and metabolic activity by impairing adhesion, reduction in surface hydrophobicity, disrupting the bacterial cell wall and membrane integrity, inhibiting extracellular polysaccharide production [62,116,117], enhancing antibiotic penetration, killing biofilm-associated cells [118], and altering bacterial morphology [116]. Given these reported mechanisms, derivatives of these compounds may exert comparable or complementary anti-biofilm effects, providing a rationale for the observed activity in the investigation of the present study. Taken together, these findings suggest that *C. salviifolius* extracts exert their antibacterial activity against *S. aureus*, including MRSA, through a multifactorial mode of action involving biofilm inhibition, partial interference with quorum sensing, and modulatory effects on efflux pump activity.

In contrast, evidence on *H. stoechas* is less established, but related studies on *H. italicum* indicate a strong antibiofilm potential, with effects associated with morphological alterations, membrane disruption, and bacterial lysis, confirming that the mode of action is likely linked to interference with adhesion and membrane integrity [119,120,121,122,123]. In fact, the *H. stoechas* extract neither inhibited violacein production in *C. violaceum* nor showed modulatory activity against efflux pumps in *S. aureus*. These results suggest that the anti-biofilm effect of *H. stoechas* extract may not be mediated by interference with bacterial communication or as EPI, instead, it probably acts via alternative antimicrobial mechanisms, such as interference with *S. aureus* adhesion.

Together, these observations highlight that multiple bioactive compounds in the extracts may be acting through complementary mechanisms to effectively prevent and disrupt *S. aureus* biofilms. Further studies are required to confirm that these are potential mechanisms underlying the antimicrobial activity of our extracts, particularly of *H. stoechas* extract, regarding the inhibition of biofilm formation.

## 3. Materials and Methods

### 3.1. Cistus salviifolius and Helichrysum stoechas Extracts

**Figure 4:**
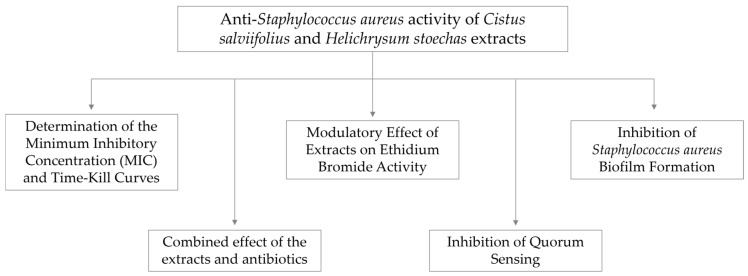
Schematic diagram illustrating the experimental design of the present work.

The wild plants *C. salviifolius* (aerial parts or stems), and *H. stoechas* (leaves and stems), were collected in the northern area of Serra da Gardunha, Portugal, during spring of 2023 (collection coordinates: 40°07′27.5″ N 7°30′22.9″ W). The plant species were identified by technicians at the Biotech Plant Lab of Beira Interior, Castelo Branco, Portugal, and a voucher specimen was deposited in our laboratory. These plants were extracted and subjected to phytochemical analysis using Ultra-High Performance Liquid Chromatography coupled with trapped ion mobility spectrometry time-of-flight mass spectrometry (UHPLC timsTOF-MS), as described previously by Coimbra et al. [41] and further characterized regarding anti-*Staphylococcus aureus* activity (Figure 4).

### 3.2. Plant Extracts, Microorganisms and Culture Media

Extracts were dissolved in dimethyl sulfoxide (DMSO, Fisher Chemical, UK) at a final concentration of 200 mg/mL and stored at −20 °C until use.

*S. aureus* reference strain ATCC 25923 and the clinical isolates MRSA 12/08 and MRSA 05/15 were grown in Tryptone Soy Broth and Tryptone Soy agar (TSB or TSA, VWR, Leuven, Belgium). The cultures were subcultured onto solid medium and incubated at 37 °C for 24 h.

### 3.3. Determination of the Minimum Inhibitory Concentration (MIC)

The susceptibility of the *S. aureus* strains to the extracts was evaluated through the microdilution method accordingly to Coimbra et al. [124]. Briefly, the inoculum, prepared by directly suspending cells in an isotonic solution of NaCl (0.85% *w*/*v*), was adjusted to a 0.5 McFarland and subsequently diluted in the TSB medium to achieve an approximate final concentration of 5 × 10^5^ colony-forming units (CFU)/mL per well. The assays were conducted using the extracts at a maximum concentration of 2000 µg/mL in a total volume of 100 µL per well. MICs for ampicillin (NzyTech, Lisboa, Portugal), ciprofloxacin (Sigma-Aldrich, St. Louis, MO, USA), and vancomycin (Thermo Scientific, Waltham, MA, USA) were also performed. After incubating at 37 °C for 24 h, 30 μL of a 0.01% resazurin solution (Sigma-Aldrich, USA) was added to each well, followed by an additional 2 h incubation at 37 °C. Inhibition of the test microorganism was indicated by the resazurin remaining blue in color. A minimum of three independent experiments, each performed in duplicate, were conducted, and the results are presented as modal values.

### 3.4. Time–Kill Curves

The time–kill curve assay with the extracts was performed based on Coimbra et al. [124] with minor modifications. Briefly, *S. aureus* strains obtained from an overnight culture were used to prepare a cell suspension with a final concentration of 10^6^ CFU/mL, and it was exposed to 0.5×, 1×, and 2× MIC of extracts. Solvent (DMSO, 1% *v*/*v*) and growth controls were also performed. Viable cell counts were determined using the drop-plate method at 0, 3, 6, 9, 12, and 24 h from the centrifuge tubes incubated at 37 °C. Each experiment was independently performed at least three times.

### 3.5. Checkerboard Assay

The checkerboard method was used to test the combined effect of the extracts and antibiotics, according to Coimbra et al. [124], with modifications. Two microplates were prepared: one in which the extract was serially diluted vertically with TSB to a final volume of 50 µL per well, and another in which antibiotics (ampicillin, ciprofloxacin, or vancomycin) were serially diluted horizontally with TSB. Subsequently, 50 µL from each well of the antibiotic plate was transferred to the corresponding well of the extract plate using a multichannel pipette. The inoculum was prepared as described in Section 3.3, and the suspension was then diluted at 1:67 with TSB, and 50 µL were added to obtain a final volume of 150 µL per well. The concentrations of the extracts and antibiotics were selected based on previously determined MIC values. The microplates were incubated at 37 °C for a period of 24 h, and after incubating, 45 μL of a 0.01% resazurin solution was added to each well, followed by an additional 2 h incubation at 37 °C. The combined effects of extracts and antibiotics were evaluated using the fractional inhibitory concentration index (FICI), calculated as the sum of the fractional inhibitory concentrations (FICs) of the extracts and the antibiotics. The FIC was defined as the MIC of the extract or antibiotic in combination divided by its MIC when used alone. A FICI value ≤ 0.5 was interpreted as indicative of synergy; 0.5 < FICI ≤ 1 as an additive effect; 1 < FICI < 4 as indifference; and FICI ≥ 4 as antagonism [67].

### 3.6. Evaluation of the Modulatory Effect of Extracts on Ethidium Bromide Activity

The modulatory effect of the extracts was evaluated using the microdilution method as previously described for ethidium bromide (Fisher Scientific, Brussels, Belgium) in the presence or absence of subinhibitory concentration (1/4× MIC) of each extract or reserpine (20 µg/mL, Sigma-Aldrich, USA), according to [83]. A positive modulatory effect was demonstrated when the presence of the extract decreased the baseline MIC of ethidium bromide. All assays were performed in duplicate across at least three independent experiments and presented as modal values.

### 3.7. Inhibition of Quorum Sensing

The inhibition of the quorum sensing by the extracts was evaluated with the biosensor strain *Chromobacterium violaceum* ATCC 12472 and performed based on Asensio et al. [125]. A bacterial suspension of *C. violaceum* was prepared from an overnight culture grown at 30 °C and 250 rpm in Luria–Bertani (LB, Liofilchem, Italy) broth and subsequently diluted in fresh LB to an optical density at 600 nm of 0.02. The extracts were serially two-fold diluted in LB, and 500 µL of each dilution was added to 48-well flat-bottom plates. DMSO at a final concentration of 0.5% was used as the solvent control. 500 µL of the bacterial suspension was added to each well, and the plates were incubated for 48 h at 30 °C. Following incubation, 750 µL were centrifuged at 5000× *g* for 3 min. The supernatants were discarded, and the pellets were vortexed with 750 µL of DMSO to solubilize the pigment violacein. Subsequently, 200 µL of the violacein-containing supernatant was transferred in triplicate to a 96-well microplate, and the absorbance was measured at 585 nm using a microplate spectrophotometer (Bio-Rad xMark, Hercules, CA, USA). After a centrifugation at 6000× *g* for 5 min, the cellular pellet was suspended in 750 µL of distilled water, and 200 µL of the suspension was transferred in triplicate to a 96-well microplate, and the optical density was measured at 600 nm.

Subsequently, the percentage of violacein inhibition (%) was then calculated using the following equation:

(1) $$Violacein\;inhibition\left(\%\right)=100-\left(\left({Abs}_{sample}/{Abs}_{growth\;control}\right)\times 100\right)$$

where Abs_sample_ represents the absorbance of each sample measured at 585 nm, and Abs_growth control_ corresponds to the absorbance of the control.

### 3.8. Antibiofilm Activity

The analysis of the effect of the extracts in biofilm formation by *S. aureus* strains was based on the previously described method of [124,126] with modifications. In brief, serial two-fold dilutions of the extracts (ranging from 0.25 to 2× MIC or 0.03 to 2× MIC, depending on the extract or strain) were prepared in TSB supplemented with 0.5% glucose in 96-well flat-bottom plates. *S. aureus* strains were cultured overnight in TSB at 37 °C with agitation at 250 rpm and suspensions diluted and adjusted to obtain a final inoculum of 1 × 10^7^ CFU/mL in each well. Subsequently, 100 µL of each bacterial suspension was added to the wells, yielding a final volume of 200 µL. The bacterial suspension in medium served as the positive control, while culture medium alone was used as the negative control. A solvent control containing DMSO 1% was also included. The plates were incubated at 37 °C for 24 h. Following incubation, the plate content was discarded, and each well was washed twice with 200 µL of distilled water to remove loosely attached cells. The adherent bacteria were then fixed with 200 µL of methanol (VWR, Rosny-sous-Bois, France) for 20 min. After removing the methanol, the plates were air-dried and subsequently stained with 0.1% (*w*/*v*) crystal violet (200 µL per well) for 10 min. Excess dye was discarded, and the wells were washed three times with 300 µL of distilled water. The bound crystal violet was then solubilized with 200 µL of 33% (*v*/*v*) glacial acetic acid, and absorbance was measured at 570 nm using a microplate reader. All assays were carried out with at least five replicates in each of three independent experiments.

### 3.9. Statistical Analysis

The statistical analysis of the results was performed using the one-way ANOVA and Dunnett test using the GraphPad Prism v8.01 software, with a 95% confidence interval, considering the values of *p* < 0.05 as statistically significant.

## 4. Conclusions

This study evaluated extracts of *C. salviifolius* and *H. stoechas* against *Staphylococcus aureus*. *H. stoechas* showed the strongest activity (MIC 7.8–62.5 µg/mL). In combination with ampicillin, ciprofloxacin, or vancomycin, *C. salviifolius* and *H. stoechas* extracts enhanced antibiotic efficacy, displaying synergistic or additive effects and restoring susceptibility of a MRSA strain. The aerial parts of *C. salviifolius* displayed modulatory effects on ethidium bromide and exhibited slight quorum sensing inhibition in *C. violaceum*, while all extracts inhibited biofilm formation even at subinhibitory concentrations. These findings highlight the dual potential of these extracts as direct antimicrobial agents and as adjuvants. Further studies are needed to elucidate mechanisms of action, specific targets, and key pharmaceutical properties such as stability and bioavailability.

## Figures and Tables

**Table 1:** Minimum inhibitory concentration (MIC, µg/mL) and minimum bactericidal concentration (MBC, µg/mL) of extracts and antibiotics on *S. aureus* strains presented as modal values.

Species	MIC (MBC)					
	*Cistus salviifolius* Aerial Parts	*Cistus salviifolius* Stems	*Helichrysum stoechas*	Ampicillin	Ciprofloxacin	Vancomycin
*Staphylococcus aureus* ATCC 25923	500 (2000)	500 (2000)	7.8 (31.3)	0.125	0.5	8
*Staphylococcus aureus* MRSA 05/15	250 (2000)	500 (>2000)	7.8 (31.3)	4	256	8
*Staphylococcus aureus* MRSA 12/08	2000 (>2000)	>2000 (ND)	62.5 (>250)	32	32	64

ND—not determined.

**Table 2:** Effect of interaction between extracts and antibiotics based on the fractional inhibitory concentration index (FICI) values. The range of FICI values obtained is presented in parentheses.

Antibiotics	*S. aureus* Strains	Extracts		
		*Cistus salviifolius* Aerial Parts	*Cistus salviifolius* Stems	*Helichrysum stoechas*
Ampicillin	ATCC 25923	Additive (≤0.98)	Additive (≤0.98)	Additive (≤0.98)
	MRSA 05/15	No interaction (≤1.06)	No interaction (1.02)	No interaction (≤1.06)
	MRSA 12/08	Synergistic (≤0.28)	Synergistic (≤0.38)	Synergistic (≤0.33)
Ciprofloxacin	ATCC 25923	Additive (≤1.00)	Synergistic (0.5)	Synergistic (0.37–0.5)
	MRSA 05/15	Additive (0.74)	Additive (0.75)	Additive (≤1.00)
	MRSA 12/08	Synergistic (0.13)	Synergistic (≤0.27)	Synergistic (0.27–0.28)
Vancomycin	ATCC 25923	Additive (1.00)	No interaction (1.01)	Additive (1.00)
	MRSA 05/15	Additive (≤1.00)	No interaction (1.01)	Additive (≤1.00)
	MRSA 12/08	Synergistic (0.50)	Additive (≤0.75)	Additive (0.57)

According to Roudashti et al. [67] a FICI value ≤ 0.5 indicates synergy, 0.5 < FICI ≤ 1 displays an additive effect, 1 < FICI < 4 denotes indifference, and FICI ≥ 4 represents antagonism.

**Table 3:** Minimum inhibitory concentration (MIC) of ethidium bromide against *S. aureus* strains in the absence (negative control) and presence of the extracts (1/4× MIC) or reserpine (positive control, 20 µg/mL).

Species	MIC (µg/mL)				
	Ethidium Bromide	+ *Cistus salviifolius* Aerial Parts	*+ Cistus salviifolius* Stems	*+ Helichrysum stoechas*	+ Reserpine
*Staphylococcus aureus* ATCC 25923	32	8	16	32	8
*Staphylococcus aureus* MRSA 05/15	32	8	32	32	8
*Staphylococcus aureus* MRSA 12/08	32	8	ND	8	16

ND—not determined. Extract concentrations of 125, 62.5, and 500 µg/mL for *C. salviifolius* aerial parts extract, 125 µg/mL were used for *C. salviifolius* stems extract, and 2, 2, and 15 µg/mL for *H. stoechas* extract, tested against *S. aureus* ATCC, MRSA 05/15, and MRSA 12/08 strains, respectively.

## Data Availability

Data is contained within the text.
